# Is low carotid bifurcation determined by vertebral level always convenient for surgical approach?

**DOI:** 10.1371/journal.pone.0294072

**Published:** 2024-02-01

**Authors:** Siriyakorn Amarttayakong, Pattama Amarttayakong, Waranon Munkong, Aroon La-up, Arada Chaiyamoon, Athikhun Suwannakhan, Sukrit Sangkhano

**Affiliations:** 1 Phu Wiang Hospital, Phu Wiang, Khon Kaen, Thailand; 2 Department of Otolaryngology, Faculty of Medicine, Khon Kaen University, Khon Kaen, Thailand; 3 Faculty of Medicine, Mahasarakham University, Mahasarakham, Thailand; 4 Department of Radiology, Faculty of Medicine, Khon Kaen University, Khon Kaen, Thailand; 5 Mahidol University, Nakhonsawan Campus, Nakhonsawan, Thailand; 6 Department of Neurosurgery, Tulane University School of Medicine, New Orleans, Louisiana, United States of America; 7 Department of Anatomy, Faculty of Science, Mahidol University, Bangkok, Thailand; 8 In Silico and Clinical Anatomy Research Group (iSCAN), Bangkok, Thailand; 9 School of Public Health, Walailak University, Nakhon Si Thammarat, Thailand; AIIMS: All India Institute of Medical Sciences, INDIA

## Abstract

Although high-level carotid bifurcation (HCB) could lead to notable surgical difficulty, the definitive reference point for HCB is unclear. HCB is typically characterized as carotid bifurcation (CB) located higher than the level of the third cervical vertebra (C), however, a major obstacle regarding carotid artery surgical exposure is angle of the mandible (AM). The objective of this study was to investigate CB level, define HCB in relation to AM and vertebral levels, and measure the vertical distance from HCB to ipsilateral AM. Moreover, the percentage of surgically challenged CBs, misclassified as low CBs (LCB) based on vertebral level, was investigated. Patients who underwent neck computed tomography angiography were retrospectively studied. HCBs were classified into two categories: CBs above the C3 and either at or above the ipsilateral AM. Of 172 CBs (86 patients; 57 men, 29 women), CB was mostly found at C3 (44.19%), whereas AM was commonly located at C2 (51.16%). Based on vertebral level and AM, HCBs were detected in 10.47% and 20.35% of CBs, respectively. The association of HCBs determined by either C3 or AM between both sides in each individual was nonsignificant (p>0.05), but HCBs determined by C3 level were predominant in women (OR = 3.58, 95%CI = 1.31–9.80). Considering both C3 and AM, there was 8.72% of HCBs. The remaining 91.28% was classified as LCBs, including 11.63% of CBs located at both C3 and AM which were actually classified as HCBs if determined by AM. In cases of CBs above AM level, the mean vertical distance was as high as 6.56 ±2.41mm.

## Introduction

Extracranial atherosclerotic disease, primarily carotid artery stenosis, is associated with a high risk of mortality and morbidity despite maximal medical therapy [1]. Carotid endarterectomy (CEA) has been revealed to reduce stroke incidence in extracranial atherosclerotic disease [1, 2]. Plaques resulting from atherosclerosis are typically found above the point of bifurcation in the carotid arteries (CB) [3]. Nevertheless, high-level carotid bifurcation (HCB) could lead to difficult surgical access during CEA to approach and treat this plaque because of restricted CB exposure [4, 5]. In the presence of HCB, adjacent structures such as the hypoglossal nerve, which provides motor innervation to the tongue [6–10], as well as the marginal mandibular, recurrent laryngeal, and accessory nerves, and the sympathetic chain [1] and submandibular gland [2], could be injured during CEA. Consequently, the "high" and "low" positions of CB, carry significant relevance as they could necessitate modifications in the suitable surgical approach, potentially influencing the choices between CEA and carotid artery stenting (CAS) [2, 11, 12].

There remains an absence of agreement on the identification of a definitive anatomical landmark that could serve as a marker for HCB. The literature offers a spectrum of definitions; many studies have described HCB as a CB that resides higher than the body of the third cervical vertebra (C3) [2, 13–15]. Alternatively, some research recommends the superior border of the thyroid cartilage [8, 16–18], the lamina of the thyroid cartilage [19], the hyoid bone [8, 16–18, 20], or a position within the first quartile of the HCB distribution within 5 cm of the mastoid process [17] as potential markers.

However, these anatomical references for defining the position of the HCB have shown limited applicability in surgical contexts [12, 17]. Indeed, some surgeons [2] and previous studies [12] have identified the angle of the mandible (AM) as a significant barrier when accessing the carotid arteries. Surgeries become notably more challenging if the CB is found at or above the level of the AM. Even with neck extension during surgery [12], the AM maintains a close anatomical position [21], leading to potential obscurity of the HCB. Therefore, alternative, more practical markers are necessary to improve surgical precision and outcomes.

Certainly, the characterization of high bifurcation should consider surgical challenges posed by structures such as the AM, a factor that currently possesses limited practical application. If HCB is determined only by vertebral level, certain CBs with surgical complications may be misclassified as low CB. Consequently, the objective of this study was to define and quantify the HCB in relation to both AM and vertebral levels, and further determine any potential misclassification of CB levels that may arise when only a single marker C3 is utilized. In addition, the vertical distance from the HCB to the ipsilateral AM was measured to gain additional anatomical information that may be beneficial for surgical planning.

## Materials and methods

### Study design and data collection

The anatomical relationship between the CB, AM, and vertebral levels was investigated through a retrospective study on patients who underwent neck computed tomography angiography (CTA) at Srinagarind Hospital, Khon Kaen University, Thailand, between January **2016** and December **2019**.

The CTA procedures were carried out using a biplane neuro-angiographic unit (Siemens Artis Zee Biplane), performing a comprehensive cerebral CTA using an 8-second 180° rotational run, injecting contrast material at a rate of 3–4 mL/s into the median cubital vein.

The study utilized a rigorous selection process, conducted by radiologist, based on the following exclusion criteria:

1. Patients exhibiting a lateral angulation of the skull >5°, neck rotation >10° or neck flexion/extension >15° [17].
2. Patients who had undergone neck surgery, interventions, or blood flow occlusion involving the common carotid artery (CCA) or its branches.
3. Patients displaying tortuosity, kinking, and coiling of the CB.
4. Patients with conditions affecting the cervical vertebrae such as fusion, Klippel-Feil Syndrome, intervertebral disc desiccation or herniation, anterior cervical discectomy and fusion (ACDF), or cervical vertebrae fracture involve in CB position.
5. Patients receiving ventilator therapy (with an endotracheal tube inserted) or having neck masses that affect CB positioning.
6. Patients under the age of 18.

The study anonymously recorded demographic data and clinical characteristics. The AM and CB levels were evaluated bilaterally, relating them to the vertebral body or intervertebral disc level in the horizontal plane. HCB was defined as a CB situated above the C3 vertebra, aligned with previous literature. The positioning of HCB in relation to AM was also explored. Vertical distances from HCB to the ipsilateral AM were measured.

Expert assessors, including a specialist referee, evaluated HCB and relevant distances using computerized measurement techniques to maintain interrater reliability.

### Statistical analysis

Data management and analysis were performed using IBM SPSS Statistics version 23. Descriptive statistics, including frequencies and percentages, were computed to summarize the demographic characteristics of the study population and the prevalence of HCBs.

To determine the association between HCBs and demographic factors, a binary regression model was employed. This statistical approach enabled the examination of significant differences in HCB characteristics across different sides (left vs. right) and sexes (male vs. female). The Pearson chi-square was used to determine the differentiation in HCB determination between using the C3 and AM landmarks. Odds Ratios (OR) with corresponding 95% Confidence Intervals (CI) were calculated to measure the strength of associations. Statistical significance was set at *p*<0.05.

## Results

**Fig 1:**
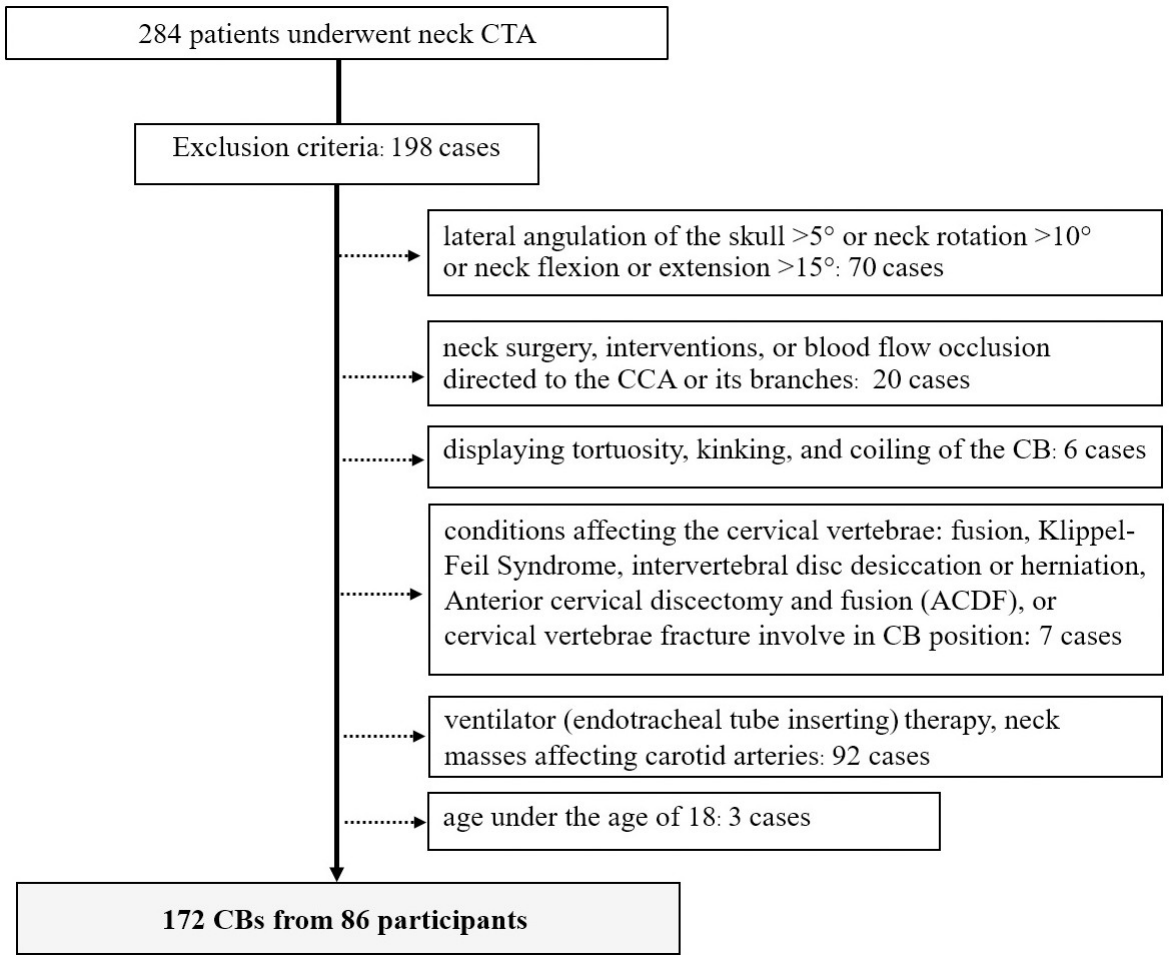
Patient selection flowchart.

In this retrospective study, we aim to determine the CB level, define HCB in relation to AM and vertebral levels, and measure the vertical distance from HCB to the ipsilateral AM. Of the 284 patients who underwent neck CTA, 86 (57 men and 29 women) met the inclusion criteria and were subsequently examined for CB level based on the CTA images (Fig 1). The average age of the participants was 51.47±18.11 (ranging from 18 to 84) years. According to CTA images, 172 CBs, (right, 86; left, 86) were investigated by Synaps software. CTA was frequently performed among patients with vascular problems (41.86%), followed by neck mass without affecting the carotid arteries (19.77%) and vertebral fracture and subluxation without affecting CB position (13.59%) (Table 1).

**Table 1:** Demographic data of the participants.

Demographic data	Value
**Sex** n (%)	
Female	29 (33.72)
Male	57 (66.28)
Total	86 (100)
**Age** mean±S.D.	
Female	52.82±15.86
Male	50.77±19.27
Total	51.47±18.11
**Clinical indication for neck CTA** n (%)	
Vascular problem	36 (41.86)
Neck mass without affecting the carotid arteries	17 (19.77)
Vertebral fracture and subluxation without effect to CBs position	12 (13.95)
Facial mass	11 (12.79)
Neck injury	8 (9.30)
No report	2 (2.33)

In the case of vascular surgical procedures in the neck and radical neck dissections, it is important to have accurate information regarding the level of the CB. Therefore, variants in CB’s neighboring structures need an examination of the anatomical distinctions. We next determine the CB and AM locations in relation to the cervical vertebral levels. In terms of vertebral level, CBs were most typically detected at C3 (44.19%), C4 (27.33%), and C3-C4 intervertebral disc (14.53%). The highest CB level corresponded to C2 (4.65%) and the lowest corresponded to C4–C5 intervertebral disc (1.74%) and C5 (1.74%) (Table 2). In the cases of AMs when compared to cervical vertebral levels, we found that most AMs were located at the level of C2 (51.16%), C3 (27.91%), and C2–C3 intervertebral disc (19.77%). The highest AM level corresponded to C2 (51.16%), whereas the lowest corresponded to C4 (1.16%) (Table 2).

**Table 2:** Distribution of the carotid bifurcation and angle of the mandible locations in relation to the cervical vertebral levels.

Cervical vertebral level	Carotid bifurcation			Angle of mandible		
	Right	Left	Total (%)	Right	Left	Total (%)
**C2**	3	5	8 (4.65)	43	45	88 (51.16)
**C2–3 intervertebral disc**	3	7	10 (5.81)	17	17	34 (19.77)
**C3**	38	38	76 (44.19)	25	23	48 (27.91)
**C3–4 intervertebral disc**	15	10	25 (14.53)	-	-	-
**C4**	24	23	47 (27.33)	1	1	2 (1.16)
**C4–5 intervertebral disc**	1	2	3 (1.74)	-	-	-
**C5**	2	1	3 (1.74)	-	-	-

In the previous results, we found that C3 can be used as the maker for HCB or LCB; however, we hypothesized that if we consider only C3, some HCBs assessed by surgical difficulty may be incorrectly categorized. Therefore, we further determined the location of CB according to C3 and AM. In Table 3, the results revealed the distribution of CB locations in relation to the levels of C3 and AM. HCBs, determined by its position above the C3 vertebra, were found in 10.47%, whereas 89.54% of the CBs were LCBs (at C3 in 44.19% and below C3 in 45.35%). Based on AM level, HCBs were found in 20.35% (above AM in 3.49% and at AM in 16.86%), whereas the remaining 79.65% were considered as LCBs.

**Table 3:** Distribution of the carotid bifurcation (CB) location in relation to the levels of the third cervical vertebra (C3) and the angle of the mandible (AM).

Location of CB	Side	Landmark	
		C3	AM
Above landmark n (%)	Right	6 (3.49)	2 (1.16)
	Left	12 (6.98)	4 (2.23)
	Both sides	18 (10.47)	6 (3.49)
At level of landmark n (%)	Right	38 (22.09)	14 (8.14)
	Left	38 (22.09)	15 (8.72)
	Both sides	76 (44.19)	29 (16.86)
Below landmark n (%)	Right	42 (24.42)	70 (40.70)
	Left	36 (20.93)	67 (38.95)
	Both sides	78 (45.35)	137 (79.65)

According to the socio-demographic data, males were found 2-fold higher that females. However, the difference of CB level between males and females were less well documented. Our study examined the association between the occurrence of HCBs based on C3 and AM levels with sides and sexes (Table 4). Analysis of the results indicated no significant difference in the association of HCBs between the left and right sides when determined by either C3 (*p* = 0.142) or AM (*p* = 0.57) levels. However, the presence of HCBs determined by the C3 level displayed a statistically significant difference between sexes (*p* = 0.013). Notably, women exhibited a higher prevalence of HCBs, with an OR value of 3.58 (95% CI = 1.31–9.80).

**Table 4:** The association between sex and side of carotid bifurcation (CB) determined by the levels of third cervical vertebra (C3) and angle of the mandible (AM).

Variable	Landmark				
	Determined by C3				
	HCB n (%)	LCB n (%)	*p-value*	OR	95%CI
**Sex**					
Female	11 (19.00)	47 (81.00)	*0.013* *	3.58	1.31–9.80
Male	7 (6.10)	107 (93.9)			
**Side**					
Right	6 (6.98)	80 (93.02)	*0*.*142*	0.46	0.17–1.3
Left	12 (13.95)	74 (86.05)			
	**Determined by AM**				
**Sex**					
Female	16 (27.60)	42 (72.41)	*0*.*096*	1.91	0.89–4.06
Male	19 (16.67)	95 (83.33)			
**Side**					
Right	16 (18.60)	70 (81.40)	*0*.*57*	0.81	0.38–1.70
Left	19 (22.09)	67 (77.91)			

*p-value<0.05

**Fig 2:**
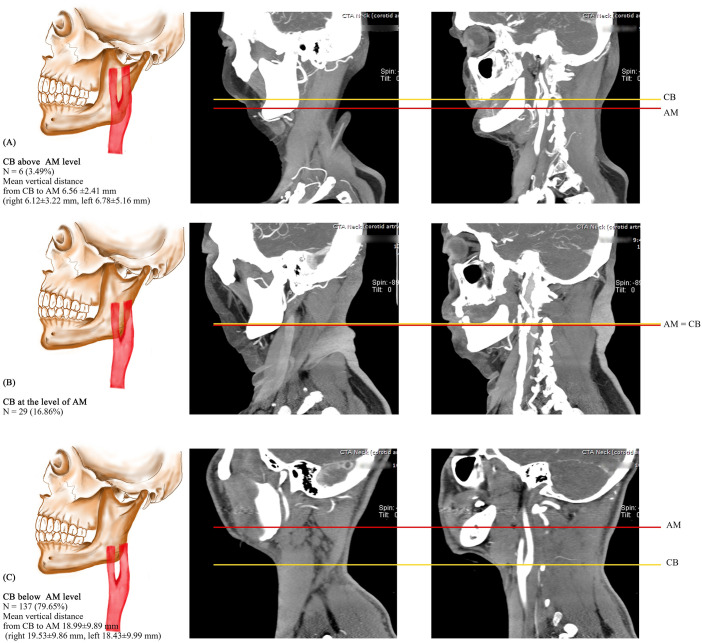
The prevalence of CB in relation to AM and the average vertical distance from CB to the AM level: (A) CB above AM; (B) CB at the level of AM; (C) CB below AM. CB: carotid bifurcation, AM: angle of the mandible.

When both C3 and AM were considered (Table 5), HCBs were found in 8.72% of the CBs (3.49% above both AM and C3, and 5.23% at AM and above C3). Utilizing C3 as the landmark, the subsequent 91.28% were designated as LCBs. It’s noteworthy that if AM had been the determinant, 11.63% of CBs located at both C3 and AM would have been classified as HCB. A statistically significant discrepancy was observed in the determination of HCB between using C3 and AM (χ2 = 49.206, *p*<0.001). This suggests that AM serves as a more efficacious landmark for pinpointing HCB. The average vertical distance from CB to AM is shown in Fig 2. In cases of CB above AM, the mean vertical distance was as high as 6.56 ±2.41mm (6.12±3.22 mm on the right side and 6.78±5.16 mm on the left) (Fig 2A). The mean vertical distance in cases with CB below AM level was up to 18.99±9.89mm (19.53±9.86 mm on the right side and 18.43±9.99mm on the left) (Fig 2C). The CB at the level of AM is presented in Fig 2B.

**Table 5:** Distribution of the carotid bifurcation (CB) considering both the levels of C3 and angle of the mandible (AM).

C3	AM		
	Above landmark	At level of landmark	Below landmark
**Right n (%)**			
Above landmark	2 (1.16)	4 (2.33)	-
At level of landmark	-	10 (5.81)	28 (16.28)
Below landmark	-	-	42 (24.42)
**Left n (%)**			
Above landmark	4 (2.33)	5 (2.91)	3 (1.74)
At level of landmark	-	10 (5.81)	28 (16.28)
Below landmark	-	-	36 (20.93)
**Both side n (%)**			
Above landmark	6 (3.49)	9 (5.23)	3 (1.74)
At level of landmark	-	20 (11.63)	56 (32.56)
Below landmark	-	-	78 (45.35)

## Discussion

Surgical strategies for patients undergoing carotid surgery essentially requires a comprehensive knowledge of the anatomical variants of CB locations [2, 12]. According to classical anatomy textbook, CB is most frequently found at the level of C3-4 [22]. However, the majority of CBs in this study were at the C3 level. It is noteworthy that the finding is consistent with a previous study comprising people of the same region (North-Eastern Thai), with the most common sites at C3 [23] (Table 6). The result was also consistent with other previous cadaveric carotid studies conducted in Japanese [24], Turkish [25], Indian [26], Kenyan [27], and Ethiopian [28] populations (Table 6). This indicates that the phenomenon might be more common than initially considered and probably is not restricted to a single population.

**Table 6:** The distribution of CB comparing with cervical vertebra level.

Authors	Method	Nationality (n)	Cervical vertebra level %									
			C1	C1-2 Intervertebral disc	C2	C2-3 Intervertebral disc	C3	C3-4 Intervertebral disc	C4	C4-5 Intervertebral disc	C5	Below C5
Anangwe et al [27]	Cadaver	Kenyan (80)	**-**	**-**	12.5	12.5	38.8	22.5	7.5	-	2.5	3.75
D. H. [28]	Cadaver	Ethiopian (26)	**-**	**-**	-	3.9	42.3	15.4	38.5	-	-	-
Zümre et al [25]	Cadaver	Turkish (40)	**-**	**-**	-	-	Rt 55	-	Rt 35	-	Rt 10	-
							Lt 60		Lt 40			
Hayashi et al [24]	Cadaver	Japanese (98)	**-**	**-**	10.2	9.1	47.5	8.0	28.4	3.4	-	-
Anu VR et al [26]	Cadaver	Indian (190)	-	-	Rt 10	-	Rt 50	-	Rt 40	-	Lt 1	-
					Lt 9		Lt 55		Lt 35			
Chaijaroonkhanarak & Prachaney, [23]	Cadaver	Thai (110)	8.2	1.8	40.0	3.6	41.8	0.9	3.6	-	-	-
Present study	CTA	Thai (172)	-	-	5.2	6.4	43	14.5	27.3	1.7	1.7	-

Considering embryology, the CCA and the proximal part of the internal carotid artery (ICA) normally originate from the third aortic arch, whereas the external carotid artery (ECA) develops from remnants of the first aortic arch with some participation from the second aortic arch [15]. Therefore, ICA is a continuation of CCA, while ECA is an anterior branch of CCA. As a result, the level of CB varies depending on where ECA originates.

Previous research indicated that AM was the most reliable landmark for evaluating the cervical level, corresponding to C2 [21], and this is consistent with the majority of AM levels in the present study. In addition, the lowest AM in our study was at C4, which was lower than those found in previous studies and could result in the CB becoming much more difficult to access in HCB cases. Most prior studies regarded HCB as CB located above the vertebral body of C3 [2, 13–15]. Based on this description, we discovered that the occurrence of HCB was as high as 10.47%, which is comparable to prior research employing the same method (CTA) in living Thai people [2], but with a lower percentage than the 19.2% observed in a Japanese cadaveric study [24].

Regarding the surgical approach, AM is considered a more suitable landmark for classifying CB lying at or above AM as HCB with high exposure challenges because AM is a significant barrier to carotid artery entrance [2, 12]. HCB determined by AM level in the present study had an occurrence of 20.35%, which is higher than the 16.50% reported by Jitpun et al [2] although our AMs were at the level of C2, which is higher than the AM observed at C2–3 intervertebral disc level in the previous report.

In cases with the CB above AM, the mean vertical distance was as high as 6.56±2.41 mm (6.12±3.22 mm on the right side and 6.78±5.16 mm on the left), which is much higher than the 0.53 mm and 0.20 mm on the right and left measured by Jitpun et al [2]. This difference indicates that although our data showed a lower HCB incidence than the previous analysis, the location was higher, indicating more exposure difficulties. Due to the inherent difficulties in surgical carotid exposure, HCBs may encourage surgeons to consider CAS instead of CEA.

In the interdisciplinary domains of human anatomy, neck surgery, and neurovascular interventions, anatomical variations have profound clinical implications. Such variations not only influence surgical methodologies but also play a pivotal role in clinical decision-making pertaining to the ideal intervention approach [29]. Our current investigation addresses an essential aspect of this subject, particularly centering on the ongoing debate concerning the most reliable anatomical landmark for delineating HCB.

Traditionally, the C3 vertebra has been employed as the gold standard landmark to differentiate between "high" and "low" carotid bifurcations [2, 13–15]. However, our data draws attention to the possible inadequacies of relying solely on the C3 as a consistent landmark. Specifically, our results indicate that approximately 12% of CBs, when assessed using AM as a reference point, would be reclassified from a low to a high bifurcation. This finding underscores the palpable clinical significance associated with anatomical discrepancies and aligns with the surgical consensus regarding the complexities encountered when the CB aligns or surpasses the AM [2, 12].

Beyond academic interest in anatomical classifications, our findings have substantial significance for clinical interventions. The decision to opt for CEA or CAS may be swayed by these anatomical subtleties [2, 11, 12]. Even if surgeons select CEA, they might determine the incision technique, such as the high transverse incision with subplatysmal flaps for the CEA method, to ensure a suitable and safe surgical approach for HCB [29]. The statistical superiority of the AM as an anatomical landmark, corroborated by our rigorous statistical analysis (χ^2^ = 49.206, *p* <0.001), lends further credence to its utility in a clinical setting. This is especially pertinent, considering the recognized challenges associated with accessing the carotid artery near the AM [2, 12].

A salient observation from our data is the gender-specific variation in HCB determination using the C3 level (*p* = 0.013) (Table 5). Remarkably, this variability is absent when the AM serves as the reference, underscoring the AM’s potential as a consistent and reliable anatomical landmark. This observation suggests that the AM might be free from some of the confounding variables that are associated with the C3 level, thereby establishing it as a more dependable marker for HCB determinations across diverse patient demographics.

However, it’s imperative to note a limitation in our study the relatively small sample size. While our findings shed light on some critical aspects of HCB determination, the sample might not be wholly representative of the broader population. That supposed, our data serves as a robust preliminary assessment, highlighting areas of clinical concern and opening avenues for more expansive research.

The implications of our research extend beyond its immediate findings. By emphasizing the clinical relevance of the AM in HCB determinations over the traditionally favored C3, we provide an evidence-backed foundation that can guide and refine surgical methodologies. Moreover, our study fills a notable gap in current literature. To the best of our knowledge, this is the first investigation exploring potential misclassifications in CBs, thereby presenting a renewed perspective against a backdrop of established views.

### Limitations

Our study, while providing valuable insights into the anatomical variations of CB and their surgical implications, has certain limitations. First, the sample size of our study was relatively small, which may impact the generalizability of our findings. Extrapolating our results to a broader population or to different geographic or ethnic groups should therefore be done. Second, the study was conducted retrospectively, using data that was not originally intended for this research. This may lead to some inherent biases and limitations related to the data.

Moreover, we used computed CTA for determining CB and the AM, which, while being a widely accepted method, might have its own limitations in terms of accuracy and precision. We also did not take into account factors like patient’s body habitus, body mass index (BMI), or specific co-morbidities, which could potentially influence the CB location and its surgical accessibility. The study was also limited by its sole focus on AM and C3 as the landmarks, which may have overlooked other significant anatomical landmarks.

### Future studies

The outcomes of our study highlight several directions for future research. First, it would be beneficial to conduct a study with a larger sample size and across multiple centers to confirm our findings and enhance their generalizability. Future studies should also consider prospective design to avoid the potential biases associated with retrospective data.

Additionally, it would be valuable to include other demographic and clinical parameters, such as BMI, body habitus, and comorbidities, to assess their impact on CB location and surgical challenges. Exploring other anatomical landmarks apart from AM and C3, and their relationship with CB location could also be of significant interest.

Given the inherent difficulties and risks associated with HCB cases, future research should explore different surgical approaches and techniques, including CAS and CEA, to determine the most effective and safest methods for such cases. Finally, it might be interesting to conduct similar studies in different populations to evaluate the influence of ethnicity and geographic location on CB location and its surgical implications.

## Conclusion

HCB is typically defined as a CB located higher than C3 vertebral level; yet AM is a significant obstacle to carotid artery surgical exposure. The present study determined HCB considering both C3 and AM levels. Our findings appear to have substantial clinical implications. CB located at both C3 and AM that should have been categorized as HCB if determined by AM might be incorrectly categorized as LCB if determined only by vertebral level. To minimize CB misclassification, both the C3 and AM markers should be examined prior to surgical planning. Due to the inherent difficulties in surgical carotid exposure, HCBs may encourage surgeons to consider CAS instead of CEA.
